# Does fact-checking COVID-19 fake news on social media correct readers’ false beliefs? A study of Chinese youths

**DOI:** 10.3389/fpsyg.2026.1593532

**Published:** 2026-08-05

**Authors:** Shuhuan Zhou, Qingyi He, Yimin Zhao, Zhian Zhang

**Affiliations:** 1 Communication University of China, Beijing, China; 2 School of New Media and Communication, Tianjin University, Tianjin, China; 3 Peking University Third Hospital, Haidian, China; 4 School of Journalism, Fudan University, Shanghai, China

**Keywords:** China, COVID-19, effectiveness, fact-checking, fake news, social media

## Abstract

**Objective:**

To examine whether fact-checking COVID-19 fake news on social media corrects false beliefs among Chinese youths, and identifies factors influencing its effectiveness.

**Methods:**

We conducted an experiment with 660 Chinese university students to compare the impact of exposure and no exposure to fact-checking on correcting misconceptions in the context of fake COVID-19 news.

**Results:**

Fact-checking significantly reduced belief in fake COVID-19 news among Chinese youths, particularly among those who initially believed it or were uncertain. In the absence of fact-checking, beliefs remained unchanged. Young people who perceived the negative impact of fake news on themselves were more receptive to fact-checking, enhancing its effectiveness.

**Conclusion:**

This study highlights the need to tailor fact-checking strategies to audience perceptions, showing that addressing perceived harm on themselves from fake news can boost acceptance of corrections. By examining fact-checking in a non-Western context, it deepens understanding of how cultural and situational factors influence the effectiveness of combating fake news.

## Introduction

The COVID-19 pandemic has killed up to 7 million people worldwide (World Health Organization, 2025). While the spread of the virus made people panic, fake news about COVID-19 also caused panic (Gupta et al., 2022; van Der Linden et al., 2020). “Fake news” is a general term used to describe inaccurate information that is often spread through social media and mimics the format of traditional news content (Greene and Murphy, 2021; Schuetz et al., 2021). It intersects with other types of information disorder, including disinformation and misinformation (Lazer et al., 2018). However, the term is widely used yet inconsistently defined across academic disciplines (Broda and Strömbäck, 2024). From the perspective of communication studies, fake news typically refers to fabricated content that mimics the style and format of legitimate news, yet lacks the editorial norms and verification practices of professional journalism (Tandoc et al., 2018). During the COVID-19 pandemic, such content often misled the public and was at times intentionally circulated through social media to achieve a conspiratorial purpose (Rocha et al., 2021). In the Chinese context, officials often label certain information as “fake news” and conduct fact-checking (Chen Q. et al., 2021; Pang et al., 2022). In this study, fake news refers to both misinformation and disinformation, encompassing verifiably false content in the form of legitimate news.

Providing readers with fact-checked information is a major means of fighting fake news, particularly in the field of medicine, including topics related to COVID-19 (Chung and Kim, 2021; Porter and Wood, 2021). In research, but also in practice, various methods of detecting the behavior in question are used, such as Information Retrieval (IR), Natural Language Processing (NLP) and Machine Learning (ML) (Zhou and Zafarani, 2020). In this study, we focus on fact-checking because, while technological detection methods are effective for large-scale fake news identification, our research involves a relatively small set of texts where verifying attribution and accuracy requires manual review (Johnson, 2024).

Many studies have found positive effects of fact-checking, such as getting people to adhere to the truth in their beliefs (Porter et al., 2018; Wood and Porter, 2019) and decreasing the public’s willingness to share fake news on social media (Freiling et al., 2021). Exposure to fact-checking can improve the accuracy of people’s beliefs (Van der Meer and Jin, 2020; Walter et al., 2020). During the COVID-19 pandemic, fact-checking has became even more crucial. Many researchers found that during the pandemic social media was rife with infodemics, including fake news, misinformation, conspiracy theories, and pseudoscientific therapies concerning the origin, diagnosis, treatment, prevention, and spread of COVID-19 (Chen and Fu, 2022; Fuchs, 2021; Naeem et al., 2021). The negative impact of the COVID-19 infodemic was more severe than in other times, causing psychological disorders, panic, depression, and fatigue among individuals (Apuke and Omar, 2021; Rocha et al., 2021). Furthermore, Due to COVID-19 fake news, many people believed the pandemic was a hoax and failed to follow public health orders and recommendations such as wearing a mask, social distancing, and getting vaccinated, increasing the spread of the virus (Tasnim et al., 2020; Zhang et al., 2021). Consequently, this infodemic undermined the effectiveness of governmental public health measures and seriously jeopardized public safety (Hartley and Vu, 2020; Ünal and Çiçeklioğlu, 2022). Fact-checking health information could help people obtain accurate information and protect themselves from COVID-19 (Burel et al., 2020; Schuetz et al., 2021).

Although fact-checking has been shown to have positive effects, scholars also note that it cannot fully eliminate the impact of fake news. Several theoretical perspectives explain these limitations, including the continued influence effect, confirmation bias, cognitive dissonance, and the illusory truth effect, all of which received significant attention during the COVID-19 pandemic. The continued influence effect highlights the persistence of COVID-19 fake news even after fact-checking, particularly when it comes from credible sources, is repeated before fact-checking, or when there is a delay between exposure and correction (van Huijstee et al., 2022; Walter and Tukachinsky, 2020). This persistence is reinforced by cognitive biases. Confirmation bias leads individuals to prefer fake news consistent with their prior beliefs, while cognitive dissonance encourages them to embrace fake news that reduces discomfort from conflicting attitudes or behaviors, such as rejecting mask mandates (Mendoza, 2023; Miller and Cabell, 2024). Repetition further compounds these processes through the illusory truth effect, where repeated exposure increases the perceived accuracy of fake news even after fact-checking (Kutscher, 2022; Udry and Barber, 2024; Vellani et al., 2023). These mechanisms together reveal the psychological and cognitive barriers that limit the effectiveness of fact-checking.

Most fact-checking research has been conducted in Western contexts, particularly the United States (Cotter et al., 2022; Schuetz et al., 2021; Zhang et al., 2021; Xue et al., 2022). In China, studies have focused on government-led fact-checking initiatives, the institutional role of fact-checking, and individual motivations to engage in it (Chen B. et al., 2021; Fang, 2022; Jiang, 2022; Lu and Shen, 2023; Wang M. et al., 2022; Wang X. et al., 2022). However, little is known about the direct impact of fact-checking on public belief change in non-Western settings. Moreover, limited research has examined audiences’ perceptions and lived experiences related to fake news, which are crucial for understanding how fact-checking messages are received and acted upon. Given cultural differences in news consumption and media trust (Humprecht, 2019), findings from Western societies may not be directly applicable to China. This gap in empirical evidence limits the development of culturally tailored fact-checking strategies for public health crises.

Given the limited evidence on fact-checking effectiveness in non-Western contexts, this study asks: (1) Does fact-checking on social media reduce belief in COVID-19 fake news among Chinese audiences without causing a backfire effect? (2) What factors influence the effectiveness of fact-checking in this context? We addressed these questions through an experiment with 660 Chinese youths randomly assigned to a fact-checking or control group, using pre- and post-tests to measure changes in belief in fake news.

## Literature review and research hypothesis

### Effectiveness of fact-checking and the “backfire effect”

As fake news spreads, providing audiences with fact-checking has become the primary way of combatting fake online news (Park et al., 2021). This is performed by official institutions, traditional media, fact-checking websites, and social media platforms (Van der Meer and Jin, 2020). The purpose of fact-checking is to correct and reduce the spread of fake news and promote the establishment of a news environment grounded in the truth (Lazer et al., 2018; Vraga and Bode, 2017a, 2017b).

Numerous studies have found positive benefits of providing audiences with fact-checking (Walter et al., 2020), including reducing people’s trust in misinformation and disinformation, persuading people to abandon extreme views, encouraging them to adhere to the truth, and decreasing the spread of false information (Brenes Peralta et al., 2022; Park et al., 2021; Porter et al., 2018). Weeks (2015) recruited 768 US adults for an online experiment and found that exposure to corrections could improve the accuracy of people’s beliefs, regardless of their emotions or political party affiliation. Subsequently, Wood and Porter (2019) designed five experiments with over 10,000 subjects. They also found that most participants accepted fact-checked information, even if it challenged their ideology, and avoided misinformation. Porter and Wood (2021) continued fact-checking experiments in Argentina, Nigeria, South Africa, and the UK. They looked at 22 different fact-checking items on various topics, such as COVID-19, local politics, crime, and the economy. They found that people are less likely to believe false information after fact-checking, and most of the effects can still be seen more than 2 weeks later.

However, some scholars have come to a different conclusion, arguing that fact-checking can have a “backfire effect.” That is, when fact-checking information contradicts people’s preexisting beliefs and attitudes, they may reject corrected information or even more strongly insist that misinformation is true (Kuklinski et al., 2000; Nieminen and Rapeli, 2019). Nyhan and Reifler (2010) did four experiments with first-year college students at a Catholic university in the US Midwest in 2005 and 2006. They had them read fake news stories with a politician’s false claim, with either a correction or no correction. They concluded that corrective information in news reports may not reduce misunderstandings and can sometimes increase them among the ideological groups most likely to hold them. This is largely because ideology can interfere with corrective effects, leading people to believe untrue statements due to political motivations (Nyhan and Reifler, 2010). This backfire phenomenon primarily involves audiences being motivated to defend their existing beliefs (Scheufele and Krause, 2019). Recently, Nyhan (2021) summarized the emerging consensus from literature research indicating that corrective information, such as fact-checking, generally results in modest but significant improvements in the accuracy of beliefs. He suggests that people often misunderstand because the corrected information is not durable, rather than due to a backfire effect. Furthermore, numerous recent studies have found that correcting misinformation increases audiences’ familiarity with it but does not affect the effectiveness of corrections (Prike et al., 2023; Swire-Thompson et al., 2022).

During the COVID-19 pandemic, some studies demonstrated that fact-checking on social media platforms can mitigate the negative impacts of COVID-19 fake news to some extent (Burel et al., 2020; Sun and Pan, 2024; Zhang et al., 2021). For instance, it helps audiences discern accurate information, protecting them from COVID-19 infection and reducing fear (Lee et al., 2023; Schuetz et al., 2021). Consequently, this study proposes the following hypothesis:

*H1*: Fact-checking effectively reduces belief in COVID-19 fake news on Chinese social media.

### Factors that influence the effectiveness of fact-checking

Many studies have explored the factors that influence the effectiveness of providing fact-checking, including the audience’s political affiliation, emotional factors, information sources, and the accuracy of the information itself (Freiling et al., 2021; Pennycook et al., 2020; Weeks, 2015; Zhang et al., 2021), but these studies have not considered audiences’ perceptions and experiences related to fake news. Since fact-checking is a correction of fake news (Walter et al., 2020), the audience’s experiences and perceptions of fake news may be important factors that affect their attitudes and behaviors toward fact-checking, which in turn directly affect the effectiveness of fact-checking (Wintterlin et al., 2021).

The more people are directly exposed to fake news, the more familiar it becomes to them. This frequent exposure may lead people to believe that fake news is trustworthy, a phenomenon known as the illusory truth effect (Begg et al., 1992; Swire et al., 2017). A “familiarity backfire” effect in response to fact-checking is thought to occur when original misinformation is repeated during the correction process (Schwarz et al., 2007). In other words, when misinformation is corrected, exposure to fake news in both the original story and the fact-checking can increase the perceived veracity of the news, thereby reducing the correction effect of fact-checking (Lazer et al., 2018; Swire-Thompson et al., 2020). Moreover, due to the continued influence effect, which refers to the persistence of fake news’s impact even after correction, users may continue to be influenced by the original false information (van Huijstee et al., 2022). For example, the first fake news in the experiment in this study was “COVID-19 on the surface of fruits and vegetables could infect people,” and the fact-checked message was “The probability of people getting COVID-19 by touching fruits and vegetables is very low.” The fact-checked information repeated words like “fruits and vegetables” and “infect,” reinforcing readers’ exposure to the terms in the original fake news. This may exacerbate people’s original misconceptions, hence the term “familiarity backfire.” Thus, this study proposes the following hypothesis:

*H2*: Seeing fake news before fact-checking could attenuate the efficacy of fact-checking compared with never having seen the fake news.

In addition to the experience mentioned above, the audiences’ perception of fake news may also impact the effectiveness of fact-checking. Academic discourse on audiences’ perception of media effects has long been dominated by literature on third-person effects. The third-person effect refers to people’s belief that the media has a greater negative impact on others than on themselves (Davison, 1983). This difference is considered to be an important factor affecting public attitudes and responses to fact-checking (Chung and Kim, 2021; Koo et al., 2021). However, many studies argue that the third-person effect cannot effectively predict public attitudes and behaviors in response to policies on harmful information and censorship (Chung and Moon, 2016; Salwen and Driscoll, 1997; Schmierbach et al., 2008). Salwen and Driscoll (1997) found that there is no significant correlation between the third-person effect and people’s attitudes toward supporting press restrictions. Schmierbach et al. (2008) argued that the third-person effect, when measured by the subtractive score, is not particularly meaningful, and its relationship with people’s attitudes seems to be a statistical artifact. Many scholars have found that compared to the third-person effect, “impact on oneself” and “impact on others” are more reliable variables influencing public attitudes and behaviors (Chen et al., 2015; Gunther and Storey, 2003; Guo and Johnson, 2020; Lo and Wei, 2002; Zhou and Zhang, 2023). This concept is referred to as “presumed media influence,” which encompasses the assumed effects on oneself and others (Gunther and Storey, 2003; Gunther et al., 2006; Neuwirth and Frederick, 2002). Specifically, it relates to people’s perceptions of the impact that negative information, such as pornography and fake news, has on themselves, others, or society (Baek et al., 2019; Lo and Wei, 2002). These perceptions may influence their attitudes and behaviors toward regulatory policies, such as censorship or fact-checking measures (Chung and Moon, 2016; Riedl et al., 2022).

Some researchers have found that people’s perceptions of the media’s impact on others can influence their attitudes and behaviors, leading them to change their original positions, such as supporting and being positively influenced by fact-checking (Barnidge and Rojas, 2014; Wu, 2023). For instance, Cheng and Chen (2021) surveyed 661 participants about fake news concerning a company that was shared on Facebook. They found that the presumed effects of fake news on others positively influenced public attitudes toward corrective actions. Wu (2023) also surveyed 1,045 Chinese adults and found that people’s presumed influence of health misinformation on others on social media was positively related to their attitude toward fact-checking. In addition, some researchers found that social media users exhibit a positive attitude toward correcting fake news when it addresses an issue that is important to themselves (Tandoc et al., 2019). Using a random-quota survey (*N* = 2,973) in Germany, Wintterlin et al. (2021) found that individuals’ positive attitudes and behaviors toward correcting distorted information on social media were positively influenced by the presumed impact of the information on themselves. However, the influence of these perceptions may be temporary, because social rewards such as likes and comments provide immediate dopamine through community recognition, which can reinforce engagement without necessarily producing lasting attitude change (Pennycook and Rand, 2019; Sherman et al., 2018).

As COVID-19 fake news emerged as a significant risk, it appeared likely that the perception fostered by this uncertainty would influence people’s attitudes toward fact-checking (Jiang, 2022). People’s positive attitudes toward fact-checking can improve the effectiveness of fact-checking. When individuals perceive that fake news has a negative impact on themselves or others, they are likely to shift their attitudes—from believing to disbelieving such news—either because they fear they are in a higher-risk social environment or out of concern for the well-being of others (Gunther, 1995; Wu, 2023). Hence, it can be assumed that youths’ attitudes toward fact-checking of fake COVID-19 news are predicted by their presumed influence of fake news. Thus, this study proposes the following hypotheses:

*H3*: The greater youths’ perception of the negative impact of fake news on themselves, the more positive the effect of fact-checking will be on them.

*H4*: The greater youths’ perception of the negative impact of fake news on their friends and family, the more positive the effect of fact-checking will be on them.

*H5*: The greater youths’ perception of the negative impact of fake news on society, the more positive the effect of fact-checking will be on them.

## Methodology

### Participants

We employed a stratified cluster sampling strategy. China was first divided into three macro-regions (eastern, central, and western) to capture regional variations in internet penetration (State Council of China, 2021). Participants were then recruited from three major higher education hubs: Guangzhou University Town in the eastern region, Wuhan University Town in the central region, and Chongqing University Town in the western region. Each site comprises multiple universities and represents distinct regional contexts in terms of internet penetration. From April 1, 2022, to June 30, 2022, Sojump,^1^ one of China’s leading professional survey companies, sent out experimental questionnaires to students in these three college towns. Students from a total of 28 universities participated in the survey. Participants could choose to enter the Sojump database and receive a reward of 20 yuan after completing the online experiment. Upon enrollment, participants were randomly assigned to one of two conditions: reading fake Covid-19 news with or without fact-checking information. The study protocol was approved by the Peking University Third Hospital Medical Science Research Ethics Committee. Participants read and signed an informed consent agreement that provided information about the purpose of the study, the researchers, and the research process. The agreement informed them that their participation was voluntary, they could withdraw from the study at any time, their personal information would remain confidential, and the data would be used solely for scientific research and academic purposes and would not be shared with anyone outside the research team.

A total of 660 students participated in the experiment, with 355 participants in the fact-checking group and 305 in the no-fact-checking group. The sample included 55.9% women (*n* = 369) and 44.1% men (*n* = 291). The majority (91.5%) of the participants were between the ages of 18 and 24. With respect to their university level, 53.3% (*n* = 352) were first- or second-year students, 38.9% (*n* = 257) were third- or fourth-year students, and 7.7% (*n* = 51) were postgraduate students.

University students, who tend to share characteristics such as age and level of education, were selected to minimize the interference of other complex factors. In addition, university students are major social media users and represent a large sector of Chinese youth. According to China’s seventh census in 2020, there are more than 218 million university-educated people in China (Xinhua Net, 2021). Currently more than half of young people in the 20–24 age group receive a college education or obtain a college degree (QQ, 2022). As of December 2021, the proportion of Chinese people who used mobile phones to access the internet reached 99.7%, and the number of online news consumers reached 771 million, or 74.7% of internet users, many of whom are young users (CNNIC, 2022). It is increasingly common for young Chinese to encounter fake news on social media (Tang et al., 2021). During the COVID-19 pandemic, the fake news that young people in China were exposed came primarily from mainstream social platforms such as WeChat (similar to Facebook) and Weibo (similar to Twitter, renamed X in 2023; Rodrigues and Xu, 2020). Young people use social media for longer durations than other groups, such as older adults, consequently experiencing greater exposure to fake news (Ray, 2021). Therefore, this study primarily focused on young people as the subject of research.

In the research, we based ourselves on published studies with high citation indices and general validity, which remain valid within the framework of rapidly developing research and retain their key findings.

### Design and procedure

This study employed a pretest–posttest experimental design with a control group and an experimental group. Upon recruitment, participants were randomly assigned to one of two conditions: (1) control group (*n* = 305), which read two pieces of fake COVID-19 news without fact-checking information; or (2) experimental group (*n* = 355), which read the same two fake COVID-19 news stories followed by fact-checking information. In both groups, participants’ beliefs about the news stories were measured before and after the intervention. The two fake news stories selected for this experiment were typical, widely circulated examples of COVID-19 misinformation on Chinese social media, chosen for their high visibility, strong public impact, and clear factual falsity confirmed by authoritative sources.

**Figure 1:**
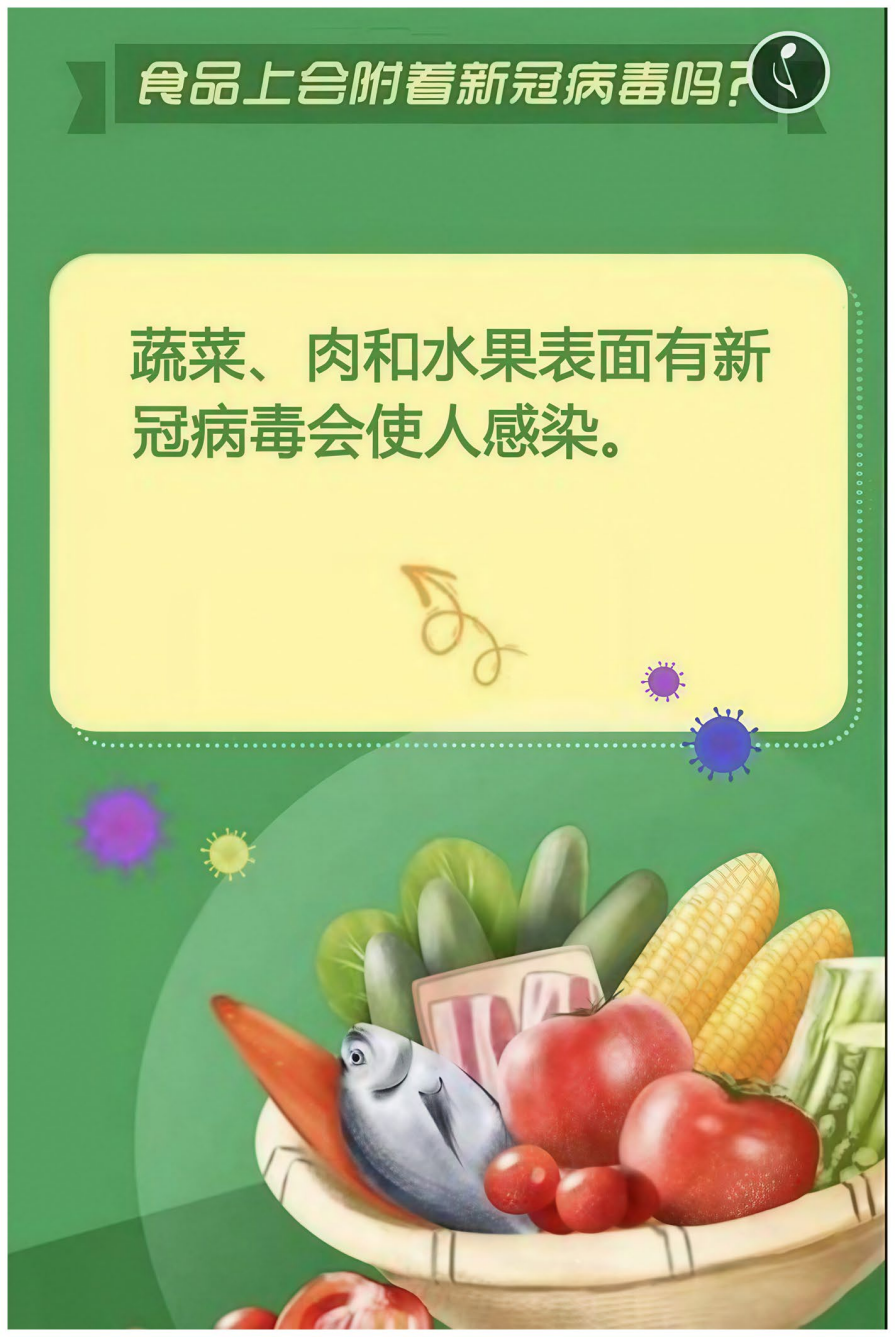
The first news story. Source: Weibo. Author-created simulated social media post used as an experimental stimulus.

The title of the first story was “COVID-19 on the surface of fruits and vegetables may infect people.” The story, which was posted on Weibo, was named one of the top 10 health rumors on social media in 2020 by a site run by a government agency called the Cyberspace Administration of China. The first fake news story (Figure 1) read:

During the COVID-19 pandemic, Weibo has been flooded with messages such as “COVID-19 on the surface of fruits and vegetables could infect people.” Because COVID-19 can survive for up to five days in the right environment, if a person with COVID-19 touches fruits and vegetables in supermarkets, the virus can spread from those fruits and vegetables to other people who touch them.

The second story, titled “Where does Omicron come from? South African scientists worry: AIDS patients may create multiple mutant strains,” appeared in Weibo’s “hot search” and was accompanied by numerous related images, including the CCTV news logo. Originating from a Phoenix.com article posted on December 8, 2021, it typifies misinformation that misinterprets or exaggerates scientific findings to provoke public fear. The story read:

Some news reports in Chinese media speculate that people might become infected with HIV when they have Omicron. South Africa has the worst AIDS crisis in the world. Nineteen percent of young people there are infected with HIV, and nearly a third of them are not receiving antiretroviral treatment. Previous COVID-19 vaccine clinical trials in South Africa showed low protection due to the association of COVID-19 with HIV infection. Low immunity may cause COVID-19 to multiply faster, promoting mutation of the virus.

#### Control group procedure

**Figure 2:**
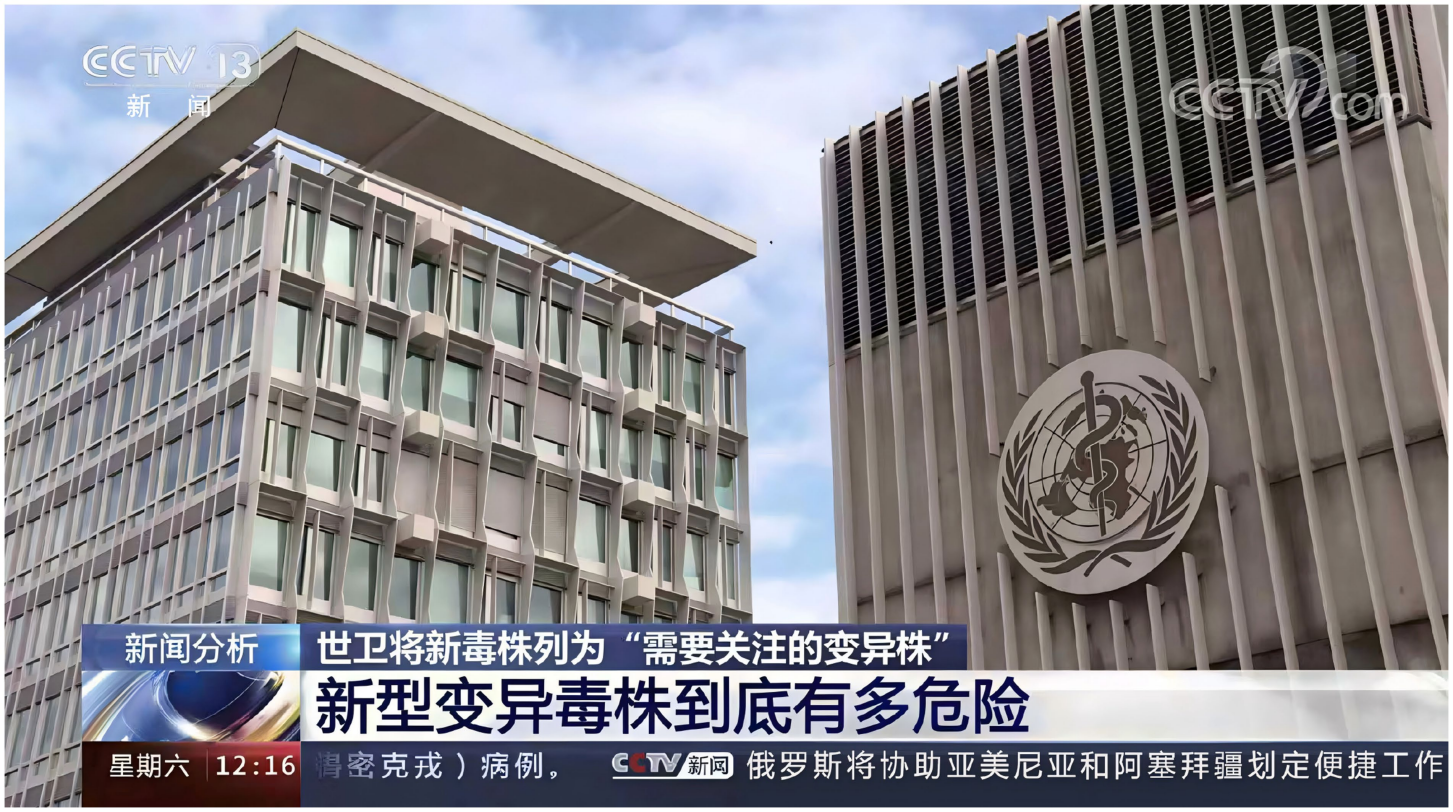
The second news story. Source: Weibo. Author-created simulated social media news stimulus used for experimental purposes.

In the pretest, participants indicated whether they had previously seen each fake news story and whether they believed it. In the posttest phase, they read an unrelated, true news story about Chinese Olympic diver Quan Hongchan’s performance at the Tokyo 2021 Olympics and then answered the same belief questions again (see Figure 2).

#### Experimental group procedure

**Figure 3:**
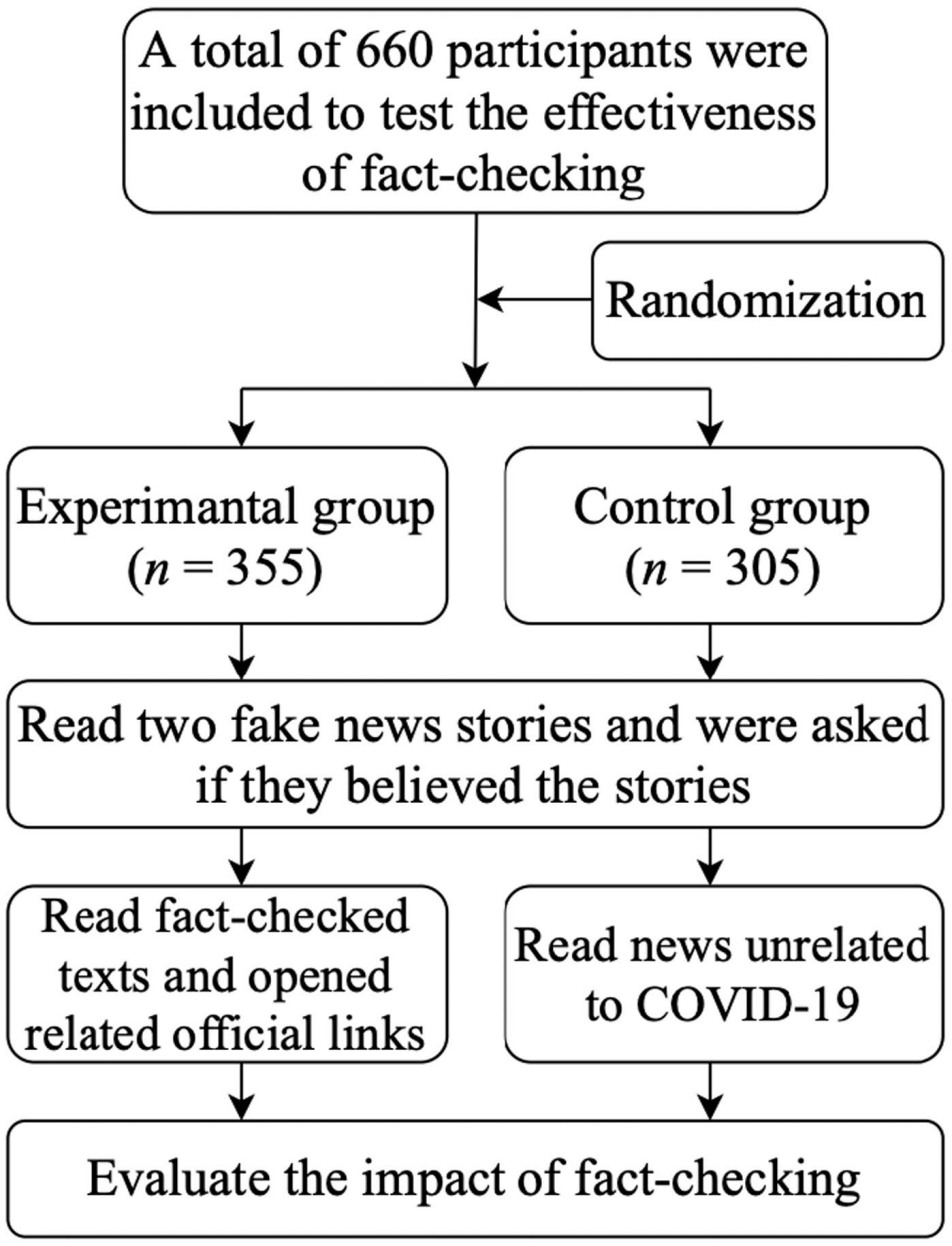
Participants’ flow of the online experiment. Source: author’s contribution.

The pretest was identical to that of the control group. In the posttest phase, participants were informed that the two stories were false. They were then presented with excerpts from fact-checking articles published by *People’s Daily and Global People Magazine*, with links to the full reports (see Appendix 1 for excerpts). All participants in this group were exposed to the fact-checking text regardless of whether they clicked on the links. After reading the fact-checking information, they answered the same questions again (Figure 3).

### Main measures

#### Fact-checking of two fake news stories

To understand young people’s attitudes toward fake news and the effect of fact-checking on them, this study designated the control group as the group without fact-checking and the experimental group as the group with fact-checking. Before and after the intervention, the control group was asked about their attitudes toward two fake news stories before and after the intervention, with the answers divided into three types: believing (true), disbelieving (false), and uncertain (Nyhan and Reifler, 2015).

For the first fake news story (Table 1), people in the control group were asked whether they believed the story (whether they thought it was true or false) before the intervention. The result was that 34.4% of participants believed it, 18.7% did not believe it, and 46.9% were not sure whether they believed it. After the intervention, the same question was asked again in the control group. The result was that 38% of participants believed it, 17.4% did not believe it, and 44.6% were not sure. When participants in the experimental group were asked whether they believed the story before the intervention, 25.1% said they believed it, 43.1% did not believe it, and 31.8% were not sure. After the intervention, 17.7% of the participants in the experimental group said they believed the story, 65.4% did not believe it, and 16.9% were not sure.

**Table 1:** Impact of fact-checking on beliefs, first fake news story.

Attitudes	Control group (*n* = 305)		Experimental group (*n* = 355)	
	Before	After	Before	After
Believe	34.4%	38.0%	25.1%	17.7%
Disbelieve	18.7%	17.4%	43.1%	65.4%
Uncertain	46.9%	44.6%	31.8%	16.9%
Stuart–Maxwell test	*χ*^2^(2) = 2.31		*χ*^2^(2) = 20.71	
	*p* = 0.315		*p*< 0.001	
Between-group difference (after) Pearson *χ*^2^(2)	129.90			
	*p*<0.001			

Source: Authors’ contribution. Within-group changes (before vs. after) were assessed using the Stuart–Maxwell test, and between-group differences after the intervention were tested using Pearson’s *χ*^2^ test.

The same procedure was repeated for the second fake news story (Table 2). In the control group, 29.8% of participants said they believed the story, 25.9% did not believe it, and 44.3% were not sure. After the intervention, 27.5% of participants said they believed the story, 29.5% did not believe it, and 43.0% were not sure. In the experimental group, 56.0% said they believed the story, 13.0% did not believe it, and 31.0% were not sure. After the intervention, 17.8% of participants in the experimental group said they believed the story, 71.5% did not believe it, and 10.7% were not sure.

**Table 2:** Impact of fact-checking on beliefs, second fake news story.

Attitudes	Control group (*n* = 305)		Experimental group (*n* = 355)	
	Before	After	Before	After
Believe	29.8%	27.5%	56.0%	17.8%
Disbelieve	25.9%	29.5%	13.0%	71.5%
Uncertain	44.3%	43.0%	31.0%	10.7%
Stuart–Maxwell test	*χ*^2^(2) = 5.80		*χ*^2^(2) = 227.64	
	*p* = 0.055		*p*< 0.001	
Between-group difference (After) Pearson *χ*^2^(2)	102.40			
	*p*<0.001			

Source: Authors’ contribution. Within-group changes (before vs. after) were assessed using the Stuart–Maxwell test, and between-group differences after the intervention were tested using Pearson’s *χ*^2^ test.

#### Effect of fact-checking

As shown in Tables 1, 2, the effect of fact-checking was calculated based on the attitudes of the participants in the experimental group (*n* = 355) before and after intervention: 0 means a negative effect, including changing from not believing the fake news story to believing it or being uncertain, or from being uncertain to believing the fake news story; 1 means that there was no change in attitude after the intervention––that is, the attitude was the same before and after the fact-checking; 2 indicates a positive effect, including changing from believing the fake news to not believing it or being uncertain about it, or from being uncertain about its veracity to disbelieving it.

#### Past experience of two fake news stories

Participants answered either “yes” or “no” when asked if they had heard the same fake news before (Chang, 2021). Those who answered “yes” were categorized as having past experience with the specific stories. For the first fake news story, 54.1% had seen it and 45.9% had not seen it. For the second fake news story, 17.5% had seen it, and 82.5% had not seen it.

#### Participants’ presumed influence of fake news on themselves

We assessed three types of participants’ presumption of the effects of fake news. The presumed influence of fake news on participants themselves was measured by three items using a 5-point Likert scale ranging from 1 = *very unlikely* to 5 = *very likely* (Baek et al., 2019; Gunther, 1995; Tang et al., 2021): (1) Fake news would make my opinion about COVID-19 more negative; (2) Fake news would make my emotions about COVID-19 more negative; and (3) Fake news would negatively affect my understanding of COVID-19 facts and events. The sum of the scores for the three items is the assigned value for the participants’ presumption of the effects of fake news on themselves. A higher score indicates that participants perceive that fake news has a greater negative impact on themselves (*M* = 8.51, *SD* = 3.34, and Cronbach’s *α* = 0.88).

#### The presumed influence of fake news on friends and family

The presumed influence of fake news on participants’ friends and family was measured by three items using a 5-point Likert scale ranging from 1 = *very unlikely* to 5 = *very likely* (Baek et al., 2019; Gunther, 1995; Wintterlin et al., 2021): (1) Fake news would make my friends’ and family’s opinion about COVID-19 more negative; (2) Fake news would make my friends’ and family’s emotions about COVID-19 more negative; and (3) Fake news would negatively affect my friends’ and family’s understanding of COVID-19 facts and events. The sum of the scores for the three items is the assigned value for the presumed influence of fake news on friends and family. A higher score indicates that participants perceive a greater negative impact of fake news on their friends and family (*M* = 11.28, *SD* = 2.43, and Cronbach’s *α* = 0.85).

#### The presumed influence of fake news on society

The impact of fake news on individuals and society varies significantly. On an individual level, fake news primarily affects psychological well-being, instilling fear and anxiety during the COVID-19 pandemic (Rocha et al., 2021). On a societal level, the repercussions are more profound, influencing public opinion and jeopardizing public health (Fernández-Torres et al., 2021). Therefore, the presumed influence of fake news on society was measured by three items using a 5-point Likert scale ranging from 1 = *very unlikely* to 5 = *very likely* (Gunther, 1995; Jang and Kim, 2018): (1) Fake news would harm society; (2) Fake news would mislead the public; (3) Fake news is undesirable in society. The sum of the scores for the three items is the assigned value for the presumed influence of fake news on society. A higher score indicates that individuals perceive a greater negative impact of fake news on society (*M* = 13.03, *SD* = 2.35, and Cronbach’s *α* = 0.92).

### Control variables

Demographics, including age, gender and education, were controlled to reduce confounding effects.

## Findings

H1 hypothesized that fact-checking effectively reduces belief in COVID-19 fake news on Chinese social media. To test within-group changes in belief distributions (Before vs. After), we conducted Stuart–Maxwell tests. As shown in Tables 1 and 2, fact-checking significantly altered belief distributions in the experimental group for both fake news stories (Story 1: *χ*^2^(2) = 20.71, *p* < 0.001; Story 2: *χ*^2^(2) = 227.64, *p* < 0.001). In contrast, no significant change was observed in the control group for Story 1 (*χ*^2^(2) = 2.31, *p* = 0.315), and only a marginal change was detected for Story 2 (*χ*^2^(2) = 5.80, *p* = 0.055). To examine between-group differences after the intervention, we conducted Pearson’s *χ*^2^ tests. The post-intervention distributions differed significantly between the experimental and control groups for both stories (Story 1: *χ*^2^(2) = 129.90, *p* < 0.001; Story 2: *χ*^2^(2) = 102.40, *p* < 0.001).

Regarding the fact-checking effect of the first fake news story, 14.1% of the participants were negatively affected by fact-checking, which was primarily a shift in attitude from being uncertain to believing fake news; 44.5% had no attitude change after the fact-checking, and 41.4% were positively affected by fact-checking, which was primarily a shift in attitude from being uncertain and believing to disbelieving fake news. Among the participants whose attitudes were the same before and after fact-checking, 11.4% believed the fake news and 76.6% did not believe it. An additional 12% of participants were unsure whether the story was true or false. Table 1 shows that 89 participants believed the fake news before the intervention. After the fact-checking, that number fell to 63, a decrease of 29.2%. After fact-checking, it fell to 60, a decrease of 46.9% from before fact-checking. Thus the fact-checking of the first fake news story had a greater impact on the unsure participants, followed by those who believed the fake news before fact-checking.

Regarding the fact-checking effect of the second fake news story, 10.1% of the participants were negatively affected by the fact-checking, 22.5% had no change in their attitude after fact-checking, and 67% were positively affected by fact-checking. As shown in Table 1, 199 participants believed the fake news before the intervention; after the fact-checking, that number fell to 63, a decrease of 68.3%. After fact-checking, it fell to 38, a decrease of 65.5%. Thus fact-checking of the second fake news story had a significant impact on the participants who believed the story initially and those who were not sure whether it was true or not. Therefore, H1 was supported.

H2 hypothesized that having previously seen a fake news story could attenuate the efficacy of fact-checking compared to never having seen it. This study used ordered logistic regression analyses to test H2 (Table 3). For the fact-checking of the first fake news story (model 1), if participants had seen it previously, the effect of fact-checking was greater (*b* = 0.36, *p* < 0.05), contrary to Hypothesis 1. For the second fake news story (model 2), past exposure did not impact the effect of fact-checking. Thus, H2 was not supported.

**Table 3:** Factors impacting the effectiveness of fact-checking.

Variables	Fact-checking of the first fake news story (model 1)		Fact-checking of the second fake news story (model 2)	
	*b*	*SE*	*b*	*SE*
Gender (1 = Female)	0.03	0.17	0.43**	0.17
Age	−0.82*	0.36	0.42	0.29
Education	1.48***	0.21	−0.12	0.14
Past experience	0.36*	0.08	−0.22	0.17
Effect on self	0.11*	0.01	0.13*	0.03
Effect on friends and family	−0.06	0.02	0.01	0.04
Effect on society	−0.002	0.02	0.01	0.03

Source: Authors’ contribution. Results are from ordered logistic regression models. Reported values are unstandardized coefficients (b) with robust standard errors (SE) in parentheses.^*^ *p* < 0.05, ^**^p < 0.01, ^***^p < 0.001.

Hypotheses 3, 4, and 5 proposed that the greater the participants’ perception of the negative impact of fake news on themselves, their friends and family, and society, the more positive the effect of fact-checking would be on them. We used ordered logistic regression analyses to test those hypotheses and explore the presumed influence of fake news on fact-checking. As seen in Table 3, for the first fake news story (model 1), only the perceived negative impact of fake news on the participants themselves was significant for predicting the effect of fact-checking. The perceived negative impact of fake news on friends and family and society had no significant impact on the effect of fact-checking. Specifically, when participants perceived a greater negative impact of fake news on themselves, the fact-checking effect was more positive (*b* = 0.11, *p* < 0.05). For the second fake news story (model 2), only the presumed influence of fake news on the participants themselves increased the fact-checking effect (*b* = 0.13, *p* < 0.05). Thus, hypothesis 3 was verified, and hypotheses 4 and 5 were not supported.

## Discussion

This study investigated the effectiveness of fact-checking on youths in China who were exposed to fake news during the COVID-19 health crisis. Through experimental research, the study found that fact-checking can have significant positive effects on young people’s attitudes toward fake news. In addition, the more participants perceived a negative impact of fake news on themselves, the greater the positive effect of fact-checking on their attitudes toward the fake news story. These results suggest that strengthening people’s perception of the negative impact of fake news on themselves, rather than on friends, family or society, through fact-checking is the key to improving the efficacy of fact-checking. This is essential for public health crisis management and emergency preparedness in order to minimize the spread of fake news that could cause people to fail to follow behaviors recommended by health and other authorities. Based on existing studies in Western countries (Liu et al., 2025; Porter and Wood, 2021), this study enriches research on the effects of fact-checking in the context of non-Western contexts. It also further extends the research on the fact-checking effect of fake news about the COVID-19 pandemic. The specific conclusions of the study are as follows.

Fact-checking significantly reduced the proportion of participants in the experimental group who believed fake news stories compared to their beliefs before the fact-checking intervention. Fact-checking had a positive effect on correcting their beliefs, compared with the control group that was not exposed to fact-checking. Specifically, young people who previously believed fake news and those who were uncertain about it disbelieved it after fact-checking. And nearly half of the participants’ attitudes remained unchanged after fact-checking the first fake news story, primarily because they had already perceived it as false and continued to regard it as fake news post-fact-checking. More than one-third of the participants were positively affected by fact-checking. This segment consisted mainly of participants who were unsure whether the story was true or false. Most participants who believed the fake news continued to believe it after fact-checking.

This shows that in some cases, fact-checking of fake news mainly changes the attitudes and positions of people who are uncertain about its veracity, while it remains challenging to correct deep-rooted misconceptions and may even increase reliance on misinformation (Buczel et al., 2024; Thorson, 2016). One reason is that when fact-checking contradicts people’s preexisting beliefs, they may resist accepting it (Kuklinski et al., 2000). Another explanation for the limited impact of fact-checking in this study is the continued influence effect, which describes the persistence of fake news even after it has been corrected. Prior research shows that fact-checking is less effective when misinformation is perceived as credible, has been repeated, or when correction is delayed (Walter and Tukachinsky, 2020). These conditions were present in our study, where COVID-19 fake news stories often cited trustworthy sources or had already circulated widely before participants encountered fact-checking. Under such circumstances, fake news may continue to shape people’s memory and judgments, making it harder for corrective information to take hold (Buczel et al., 2024; van Huijstee et al., 2022). This persistence helps explain why fact-checking in our findings was more effective for those uncertain about the accuracy of the claims, but struggled to neutralize entrenched misconceptions.

In contrast, more than two-thirds of participants were positively affected by fact-checking of the second fake news story, including those who had previously believed the story and those who were initially unsure about the authenticity of the story. The variation in the impact of the two different fake news stories on participants’ belief or disbelief may be related to the content or information sources of the stories (Guillory and Geraci, 2013). The second fake news story bore the logo of CCTV (the largest state-run TV channel in China), which the participants may have viewed as a credible news source, leading them to believe the story before fact-checking. This also demonstrates the high level of trust Chinese people place in state media and the influence of authoritative sources on the effectiveness of fact-checking (Walter and Tukachinsky, 2020; Xu, 2013). We acknowledge that, although debunking typically relies on the authority of credible media institutions, these organizations may have fake news (Nelson, 2016).

This study found that the main factor influencing the effect of fact-checking is that the participants perceived a negative impact of fake news on themselves, but not on their friends, family and society. The greater their perceived negative impact of fake news on themselves, the more fact-checking positively affected their attitude change. This validates Wintterlin et al.’s (2021) finding that people react more positively in fighting distorted information only when they perceive it to impact themselves, because they only care about and are willing to correct fake news related to themselves (Tandoc et al., 2019). Thus, when people perceive fake news to have a greater negative impact on themselves, they are more ready to change their attitude from belief or incertitude to disbelief after fact-checking to protect their personal welfare (Gunther, 1995).

We found differences in the effect of past experience with specific fake news stories on the effectiveness of fact-checking. Participants who had seen the first fake news story previously were more likely to be positively influenced by fact-checking than those who had never encountered it. This finding is inconsistent with the familiarity backfire effect described by other scholars (Ecker et al., 2020; Thomas and Autry, 2024; Wood and Porter, 2019). According to past research, fact-checking will reinforce the audience’s familiarity with misinformation, such that their belief of fake news is strengthened––the opposite effect of the intended impact of fact-checking (Begg et al., 1992; Swire et al., 2017; Udry & Barber,2024). In our experiment there was a short interval between participants’ reading fake news and fact-checking. The rapid fact-checking may have overcome any reinforcement of their familiarity with the stories, such that the fact-checking was effective (Barbetta et al., 1994; Walter and Tukachinsky, 2020). Thus past experience did not impact the effect of fact-checking. This confirms recent research conducted by several scholars in the Chinese context that found that improving familiarity does not impact the effectiveness of fact-checking, but repeating certain information can improve the accuracy of public beliefs and increase the effectiveness of fact-checking (Nyhan, 2021; Prike et al., 2023; Wahlheim et al., 2020). The reason for different influences of past experiences on the two types of fake news fact checks may be related to the type of fake news. The first fake news story was more directly related to participants’ health and well-being, and thus may have had a greater positive impact on those who had encountered the news previously. In contrast, the second story pertained more to scientific knowledge of the pandemic and thus may have had less impact on the participants.

## Theoretical and practical implications

Theoretically, this study makes three key contributions. It deepens the understanding of fact-checking effectiveness by integrating the continued influence effect to explain why fact-checking fails for some individuals, while confirming that it does not cause a “backfire effect” in the Chinese social media context. The research also extends the “presumed media influence” theory by demonstrating that fact-checking’s effectiveness is driven by an individual’s perception of a direct negative impact on themselves, rather than on others. Practically, the study’s findings could provide a basis for varied fact-checkers, illustrating the effectiveness of fact-checking in debunking false beliefs among public. Furthermore, fact-checkers could enhance the efficacy of their efforts by improving the public’s awareness of the negative influences of fake news, viewing the public as an active contributor to the problem (Boudana and Segev, 2026).

## Limitations

This study has the following limitations. First, the participants were university students, who may not be fully representative of the broader youth population or society as a whole. Future research should include more diverse samples covering different educational backgrounds, occupations, and age groups to improve representativeness. Second, we used an experimental method to study how fact-checking influences attitudes toward fake news. A real-world, longitudinal, or field study may yield different results by capturing more complex dynamics of exposure, sharing, and correction. Third, this study utilized only two fake news stories for experimentation. In future research, a broader selection of fake news types should be employed to enhance the robustness and generalizability of the findings. Fourth, this study selected pandemic-related fake news because fact-checking is generally aimed at fake news about urgent topics. When considering the influencing factors of fact-checking, our study only analyzes factors such as people’s perception and exposure to fake pandemic news from the perspective of the audience. Future research could broaden this scope by investigating how textual features, credibility of information sources, and characteristics of communication channels interact with audience factors to influence the impact of fact-checking. Finally, while the experimental design strengthens causal inference regarding the manipulated factor, the regression-based tests of H3–H5 rely on contemporaneously measured perception variables. Therefore, these interaction effects should be interpreted with caution, as the temporal ordering between perceived negative impact and the effectiveness of fact-checking cannot be definitively established. Future research employing longitudinal designs or experimental manipulation of perceived risk would help provide stronger evidence for causal inference.

## Data Availability

The raw survey data supporting the conclusions of this article will be made available by the authors upon reasonable request, after removal of all identifiable personal information to protect participants’ privacy, without undue reservation.

## Footnotes

1
http://www.sojump.com

## Appendix 1: fact-checking texts

### Story #1, from the *People’s Daily*, one of China’s mainstream official media

“During the COVID-19 pandemic, Feng Luzhao, a researcher at the Chinese Center for Disease Control and Prevention, stated that the likelihood of the COVID-19 virus contaminating vegetables, meat, and fruits through droplets or direct contact is very low. The virus can be killed in 30 min at a temperature of 56 °C, whereas normal cooking temperatures can reach 100 °C or higher. By washing and cooking food thoroughly, as well as washing hands before eating and after using the restroom, we can prevent the virus from spreading through the digestive system” (Source: http://m.people.cn/n4/2020/0213/c204447-13681169.html).

### Story #2, from *Global People Magazine*, issue 549, also one of China’s mainstream official media

“A reporter interviewed Wu Guizhen, the chief expert at the Institute of Viral Disease Prevention and Control of the Chinese Center for Disease Control and Prevention. Wu stated, ‘It is completely baseless to worry about simultaneous infections of Omicron and HIV; the two are not related. Currently, just over 20% of South Africa’s population has been fully vaccinated against COVID-19. Given that South Africa has a high rate of HIV infection, generally low immunity, low levels of COVID-19 vaccination, and limited protection, these factors collectively contribute to the easier replication of the COVID-19 virus and the emergence and spread of mutant strains. Being infected with Omicron is not linked to an HIV infection’” (Source: https://baijiahao.baidu.com/s?id=1718104393731962191).

## Appendix 2

**Table A1:** Balance tests for randomization.

Characteristic	Control (*n* = 305)	Experimental (*n* = 355)	*χ*^2^ (df)	*p*
Gender			*χ*^2^(1) = 0.01	0.94
Female	171 (56.1%)	198 (55.8%)		
Male	134 (43.9%)	157 (44.2%)		
Age			*χ*^2^(1) = 0.03	0.87
18–24	278 (91.1%)	327 (92.1%)		
Other	27 (8.9%)	28 (7.9%)		
University Level			*χ*^2^(2) = 0.02	0.99
First/Second-year	163 (53.4%)	189 (53.2%)		
Third/Fourth-year	118 (38.7%)	139 (39.2%)		
Postgraduate	24 (7.9%)	27 (7.6%)		
